# Health conditions in adults with atrial fibrillation compared with the general population: a population-based cross-sectional analysis

**DOI:** 10.1136/heartjnl-2024-324618

**Published:** 2025-06-13

**Authors:** Thomas J. Downes, Bruce Guthrie, David Moreno-Martos, Daniel R. Morales

**Affiliations:** Division of Population Health and Genomics, University of Dundee, Dundee, UK; Advanced Care Research Centre, Usher Institute, University of Edinburgh, Edinburgh, UK; Division of Population Health and Genomics, University of Dundee, Dundee, UK; Division of Population Health and Genomics, University of Dundee, Dundee, UK

## Abstract

**Background:**

Atrial fibrillation (AF) prevalence is rising due to population ageing and comorbidity is an increasing problem. The aim of this study was to examine the prevalence and association of co-existing health conditions among adults with AF in the general population.

**Methods:**

Cross-sectional analysis of Clinical Practice Research Datalink (CPRD) primary care electronic medical records in England linked to hospital admissions as of 30/11/15. CPRD is broadly representative of the UK general population in terms of age, sex and ethnicity. We estimated prevalence and used logistic regression examining risk factors of age, sex and socioeconomic status (SES) to compare prevalence of 252 physical and mental health conditions and 23 higher-level health condition groups in adults with AF compared to adults without.

**Results:**

34,338 adults with AF (57% male; 83% ≥65 years) and 907,739 without AF (49% male; 23% ≥65 years) were identified. Adjusted for age and sex, adults with AF were significantly more likely to have 20/23 (87%) health condition groups than adults without AF. The most prevalent health condition groups in adults with AF were cardiovascular (prevalence 89% in adults with AF vs 26% in adults without, adjusted odds ratio (aOR) 5.82, 95%CI 5.60-6.05), gastrointestinal (62%vs37%, aOR 1.34, 95%CI 1.31-1.38) and orthopaedic (58%vs24%, aOR 1.32, 95%CI 1.29-1.35). 151/252 individual conditions were significantly more common in adults with AF including cardiovascular conditions such as cardiomyopathy (4.5%vs0.3%, aOR 9.58, 95%CI 8.88-10.35) and heart failure (18%vs0.7%, aOR 9.07, 95%CI 8.70-9.46), and non-cardiovascular conditions such as pleural effusion (16%vs1.8%, aOR 3.55, 95%CI 3.42-3.67) and oesophageal malignancy (0.3%vs0.0%, aOR 2.14, 95%CI 1.69-2.70). Associations were similar after SES adjustment.

**Conclusions:**

Whilst cardiovascular conditions are highly prevalent and strongly associated with AF, a wide spectrum of non-cardiovascular conditions were also strongly associated requiring a greater understanding of managing comorbid conditions with management principles contradictory to AF.

## Introduction

Atrial fibrillation (AF) is a common arrythmia associated with a high lifetime risk of heart failure, stroke and myocardial infarction [1]. The global prevalence of AF is increasing, having more than doubled between 1990 and 2019 to nearly 60 million cases [2]. Whilst survival in adults with AF has increased, a mortality gap compared with adults without AF remains, highlighting the ongoing importance managing risk factors and comorbidity to improve outcomes [3].

Comorbidities are associated with an increased rate of hospitalisation and all-cause mortality in adults with AF [4, 5]. Whilst AF incidence and prevalence increases with age, modifiable risk factors including hypertension and excess alcohol consumption have been shown to vary by socioeconomic status (SES) [6, 7]. Timely identification and management of comorbid diseases remain integral parts of the management of AF with the 2024 European Society of Cardiology (ESC) guideline on the management of AF placing comorbidity and risk factor management at the forefront of its management principles [8]. Whilst existing AF guideline recommendations refer to a select number of well-known comorbidities, a comprehensive understanding of comorbidity more broadly, is limited. For example, population-based studies [7, 9, 10] have typically examined limited numbers of conditions and seldom consider acute precipitants such as infections, that have been attributed to nearly 20% of newly diagnosed AF cases [11].

Comprehensively understanding comorbidity in AF more broadly helps in a number of ways including supporting clinicians and policy makers better anticipate service needs and provide holistic care, understanding target AF populations in routine care to judge the generalisability of clinical trial evidence, and identifying less characterised or even new disease-disease interactions requiring further research. The aim was therefore to perform a large-scale characterisation measuring the prevalence of comorbidity more broadly in adults with and without AF.

## Methods

### Data source

The UK Clinical Practice Research Datalink (CPRD) GOLD database contains anonymised data from electronic primary care records for ~7% of the UK population [12]. CPRD GOLD has demonstrated high validity [13] and people are broadly representative of the UK general population in terms of age, sex and ethnicity [12]. Primary care data contained patient demographics, including age and sex, Read codes for diagnoses, and laboratory tests of renal function and lipid profile. CPRD GOLD data from England were linked to the Hospital Episodes Statistics (HES) [14] to identify additional diagnoses and procedural codes from people admitted to NHS hospitals in England using the international classification of diseases, tenth revision (ICD-10) codes. SES was measured using the Index of Multiple Deprivation (IMD) (2015) for which data were derived through linkage with the UK Office of National Statistics (ONS). The IMD ranks 32,844 small areas in England by relative deprivation [15]. Patient-level IMD data was categorised into deciles of the English population and practice-level data was used if patient-level was unavailable. Study approval was granted by the independent scientific advisory committee of the Medicines and Healthcare products Regulatory Agency (20_018). Informed consent was not required for this study.

### Study design and population

A cross-sectional study design was used [16] including adults aged ≥18 years registered for at least two years at general practices contributing up-to-standard linked data to CPRD GOLD as of the cross-sectional index date (30^th^ November 2015).

### Atrial fibrillation

Adults with AF were identified using Read (Supplementary table 1) and HES ICD-10 (Supplementary table 2) codes (recorded in any position in the discharge record) for AF and atrial flutter recorded any time prior to the index date.

### Health conditions and condition groups

The occurrence of 252 health conditions was measured both individually and as 23 higher-level groups containing two or more conditions. Comorbidities were identified using Read and ICD-10 codes recorded any time prior to the index date. Included health conditions were selected from an established list of 308 frequent physical and mental health conditions with previously defined codelists [17]. Perinatal conditions (n=14) and conditions deemed unsuitable for inclusion (n=8; Supplementary table 3) were excluded, and similar clinical entities were combined (n=50 were combined into 17 aggregate conditions, Supplementary table 4).

Chronic kidney disease (CKD) was additionally defined biochemically using the most recent blood test value prior to the index date, as an estimated glomerular filtration (eGFR) rate of <60 ml/min/1.73m^2^. Abnormal lipid levels were defined using most recent blood test value using the following cut-offs: total cholesterol (TC) >5 mmol/l; low-density lipoprotein (LDL) cholesterol >3 mmol/l; high-density lipoprotein (HDL) cholesterol <1 mmol/l; and triglycerides (TG) >2.3 mmol/l. A person was assumed to not have a diagnosis when a code or test was not recorded in their health record. The proportions of adults with lipid blood test results are shown in Supplementary table 5.

### Statistical analysis

Age, sex and IMD between adults with and without AF were compared, with the Wilson score interval used to calculate exact 95% confidence interval (95%CI) for differences. The proportion of adults with each health condition and condition group recorded prior to the index date were calculated amongst adults with and without AF. Logistic regression was used to calculate odds ratios (OR) for the likelihood of each health condition and condition group in adults with AF compared to adults without AF adjusting first for risk factors of age (continuous variable) and sex (binary variable), and then for age, sex and IMD to assess the impact of SES as a risk factor, using R version 4.1.3 [18].

### Patient and public involvement

Patients were not involved in the design, conduct or dissemination plans of this study.

## Results

**Figure 1:**
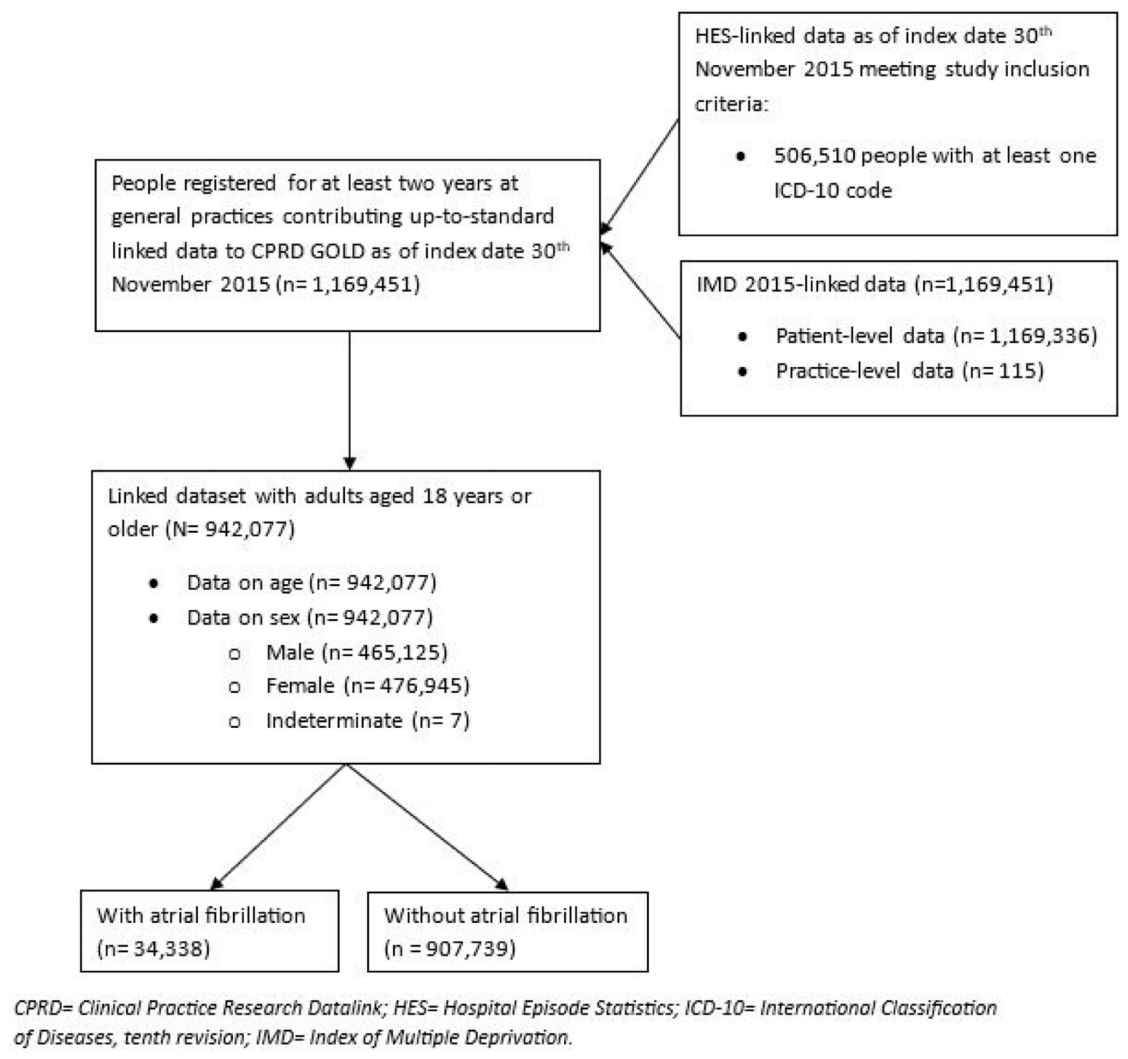
Selection of people to study sample.

Of 942,077 adults included in the cross-section, 34,338 (3.6%) had a diagnosis of AF (Figure 1). Population characteristics are shown in Table 1, with each variable containing complete data. Compared to adults without AF, a greater proportion of adults with AF were men (57% vs 49%) and were older (83% vs 22% aged 65 years and older), whilst quintile SES percentage was similar.

### Condition groups

**Figure 2:**
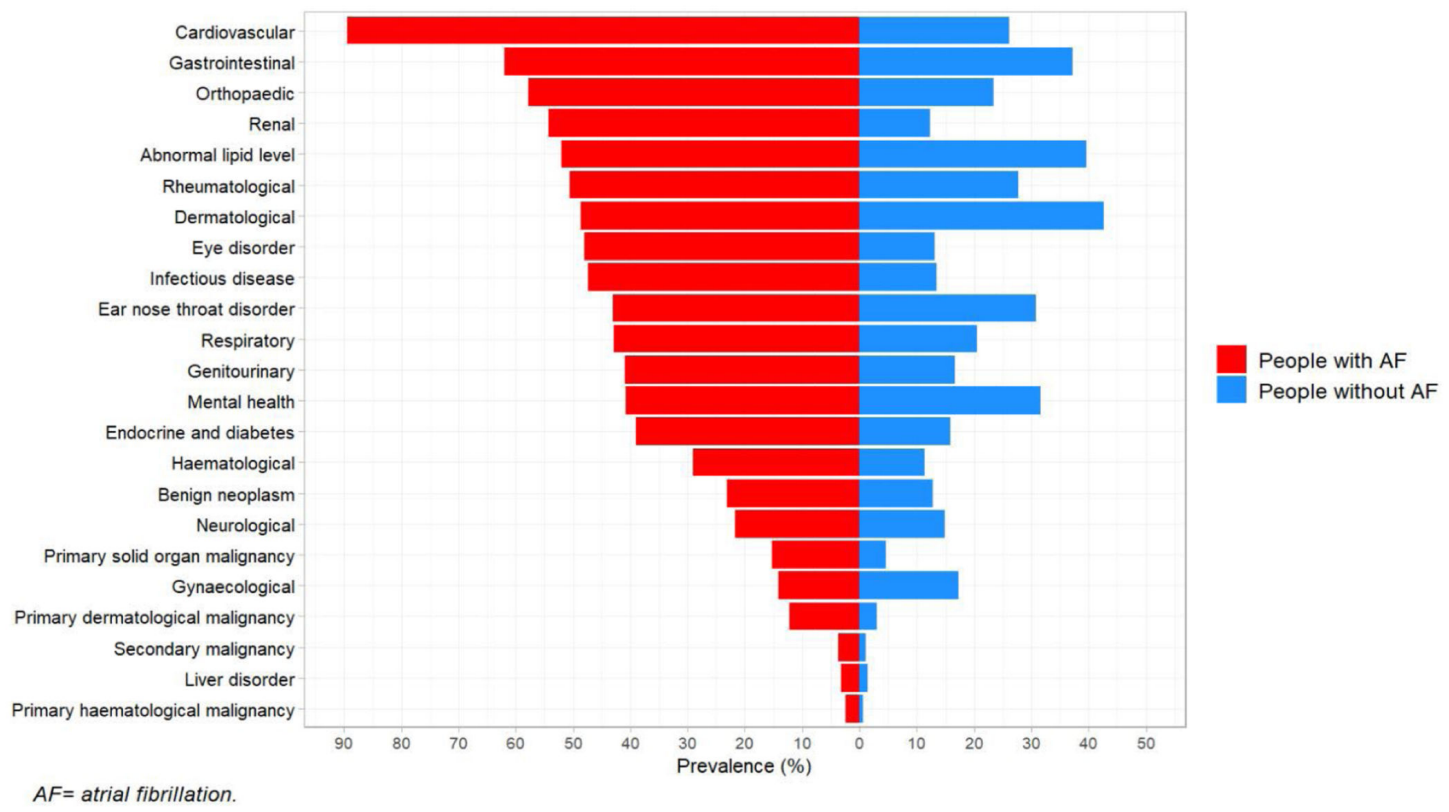
Prevalence of health condition groups in adults with and without atrial fibrillation.

The prevalence of condition groups in adults with and without AF are shown in Figures 2 and Table 2. The most common condition groups in adults with AF compared to those without AF were cardiovascular (89% vs 26%), gastrointestinal (62% vs 37%), orthopaedic (58% vs 24%), renal (54% vs 12%), lipid (52% vs 40%) and rheumatological (50% vs 28%) conditions, with eight other condition groups occurring in 30-49% of adults with AF. In contrast, only gynaecological conditions were more prevalent in adults without AF (14% vs 17%).

**Figure 3:**
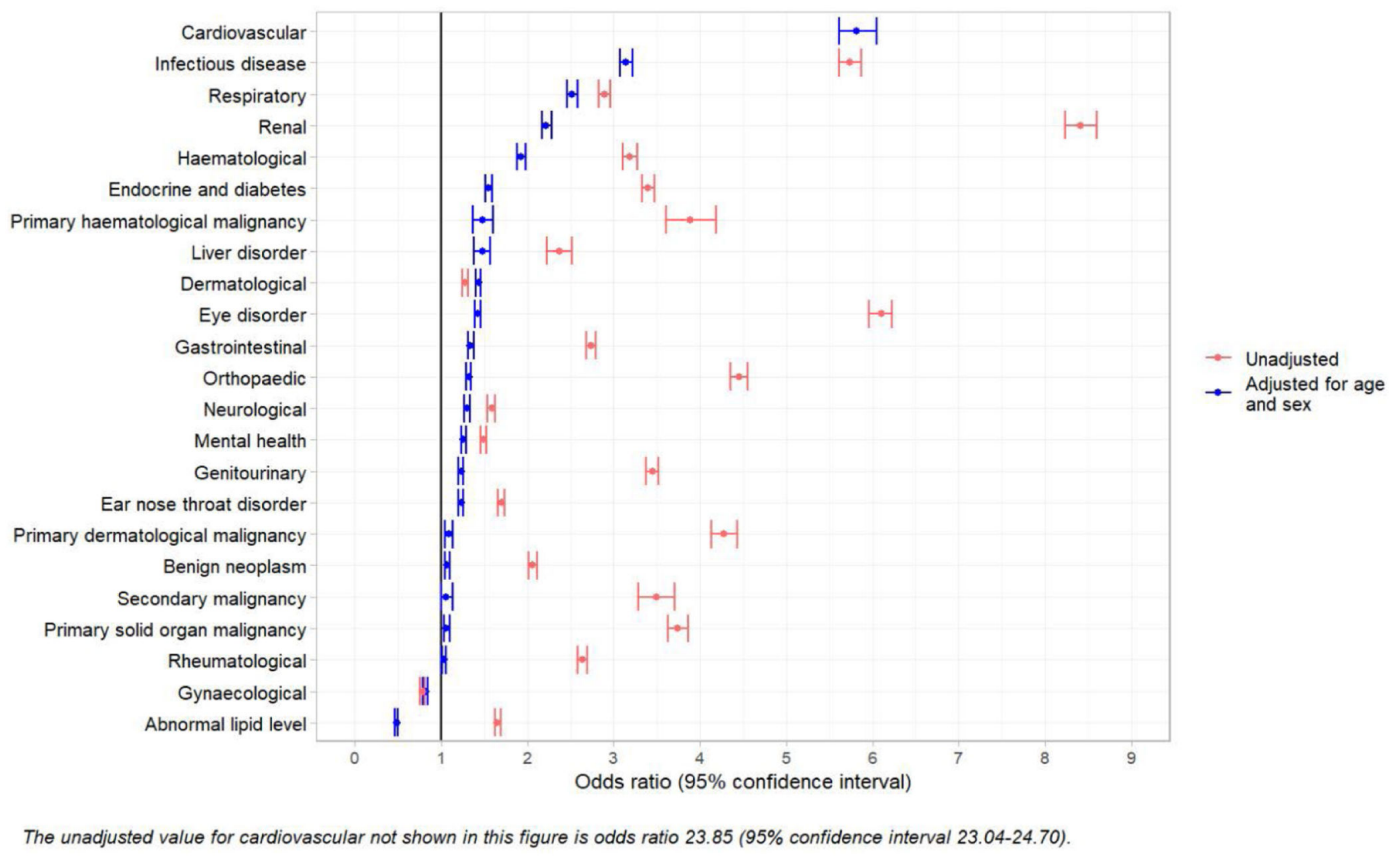
Odds ratio for health condition groups in adults with atrial fibrillation compared to adults without.

After adjustment for age and sex, adults with AF had statistically significantly higher odds of having 20 out of 23 (87%) conditions groups than adults without AF (Figure 3). The strongest associations were observed with cardiovascular (adjusted OR [aOR] 5.82, 95%CI 5.60-6.05), infectious (aOR 3.14, 95%CI 3.07-3.22), respiratory (aOR 2.52, 95%CI 2.46-2.58) and renal (aOR 2.22, 95%CI 2.17-2.28) conditions.

### Individual conditions

#### Cardiovascular

As expected, individual cardiovascular conditions were highly prevalent in adults with AF compared to those without, including hypertension (78% vs 23%), coronary heart disease (45% vs 6.2%), stroke (18% vs 2.1%) and heart failure (18% vs 0.7%). Following adjustment for age and sex, the strongest associations were observed for sick sinus syndrome (aOR 10.94, 95%CI 9.79-12.23), cardiomyopathy (aOR 9.58, 95%CI 8.88-10.35) and heart failure (aOR 9.07, 95%CI 8.70-9.46) (Table 3).

The individual non-cardiovascular conditions most prevalent in people with AF are shown in Table 4.

#### Endocrine and lipid

Highly prevalent endocrine or lipid conditions in adults with AF compared to adults without included raised cholesterol (31% vs 31%), raised LDL cholesterol (29% vs 26%), type 2 diabetes mellitus (19% vs 5.7%), low HDL cholesterol (17% vs 7.9%) and thyroid disease (16% vs 6.0%). All endocrine conditions, except type 1 diabetes mellitus, had a higher odds in adults with AF (Supplementary table 6). Adults with AF had higher odds of low HDL cholesterol (aOR 1.33, 95%CI 1.29-1.38) and lower odds of raised triglycerides (aOR 0.79, 95%CI 0.76-0.82), raised LDL cholesterol (aOR 0.44, 95%CI 0.43-0.46) and raised cholesterol (aOR 0.36, 95%CI 0.35-0.37).

#### Mental health and neurological

The most prevalent mental health or neurological conditions in adults with AF compared to adults without included depression (21% vs 21%), dementia (11% vs 1.4%), peripheral or autonomic neuropathy (7.3% vs 2.6%) and alcohol problems (6.7% vs 3.9%). Of the 27 mental health or neurological conditions, 13 had higher odds and five had lower odds in people with AF compared to those without (Supplementary table 7). Conditions with the strongest associations were Autism and Asperger’s syndrome (aOR 2.56, 95%CI 1.76-3.60), delirium (aOR 2.28, 95%CI 1.97-2.63) and subdural haematoma (aOR 2.28, 95%CI 1.99-2.62).

#### Respiratory, gastrointestinal and liver

The most prevalent respiratory or gastrointestinal conditions in adults with AF compared to adults without were gastro-oesophageal reflux disease, oesophagitis and oesophageal ulcer (24% vs 12%), abdominal hernia (19% vs 7.4%), pleural effusion (16% vs 1.8%) and asthma (15% vs 15%) (Supplementary table 8). Most respiratory (11/13), gastrointestinal (14/19) and liver conditions (3/5), had a higher odds in adults with AF compared to adults without, with pulmonary hypertension (aOR 8.61, 95%CI 7.97-9.30), pleural effusion (aOR 3.55, 95%CI 3.42-3.67) and pulmonary embolism (aOR 2.24, 95%CI 2.10-2.39) most strongly associated with AF.

#### Orthopaedic and rheumatological

The most prevalent orthopaedic or rheumatological conditions in adults with AF compared to adults without included osteoarthritis (44% vs 14%), enthesopathies and synovial disorders (37% vs 23%) and fracture of hip, wrist or vertebra (15% vs 5.0%). Of the orthopaedic or rheumatological conditions, 10/23 had a higher odds and 4/23 had lower odds in adults with AF (Supplementary table 9).

#### Genitourinary, gynaecological and renal

The most prevalent genitourinary or renal conditions in adults with AF compared to adults without included CKD (41% vs 7.7%), acute kidney injury (AKI) (24% vs 2.8%) and hyperplasia of prostate (18% vs 3.6%) (Supplementary table 10). Following adjustment, the odds of all renal and most genitourinary (5/7) conditions were increased in adults with AF, with the strongest associations observed with AKI (aOR 2.76, 95%CI 2.68-2.85) and tubulointerstitial nephritis (aOR 2.57, 95%CI 2.25-2.92). Most gynaecological conditions (8/9) had lower odds in adults with AF including menorrhagia and polymenorrhoea (aOR 0.51, 95%CI 0.48-0.54) and cervical intraepithelial neoplasia (aOR 0.56, 95%CI 0.47-0.67).

#### Special senses, haematological conditions and Down’s syndrome

Special senses conditions highly prevalent in adults with AF compared to adults without included cataract (34% vs 7.0%), dermatitis (31 vs 26.0%) and hearing loss (24% vs 9.1%). Of the special sense conditions, 13/30 had higher odds and 5/30 had lower odds in people with AF and there was no difference in odds of Down’s syndrome (aOR 0.52, 95%CI 0.19-1.15) (Supplementary table 11). The odds of all haematological conditions, except sickle cell anaemia and thalassaemia, were increased in people with AF and the strongest associations observed with immunodeficiencies (aOR 2.75, 95%CI 1.85-3.98) and thrombocytopaenia (aOR 2.42, 95%CI 2.24-2.61) (Supplementary table 12).

#### Benign neoplasms and malignancies

Benign neoplasms or malignancies more prevalent in adults with AF compared to adults without included primary malignancy other skin and subcutaneous tissue (11% vs 2.7%) and benign neoplasm of colon, rectum, anus, and anal canal (10% vs 3.5%). Of the benign neoplasms and malignancies, 7/48 had lower odds and 20/48 had higher odds in adults with AF (Supplementary table 13). The strongest associations were observed with primary oesophageal malignancy (aOR 2.14, 95%CI 1.69-2.70) and primary malignancy of bone and articular cartilage (aOR 2.04, 95%CI 1.18-3.34).

#### Infectious diseases

Infectious diseases recorded most frequently in adults with AF compared to those without included other infection (32% vs 7.6%), bacterial diseases (excluding tuberculosis) (27% vs 5.9%) and urinary tract infections (25% vs 4.5%). Most (21/23) of the infectious diseases had a higher odds in adults with AF (Supplementary table 14). The strongest associations were observed for infections of the heart (aOR 7.40, 95%CI 6.09-8.98) and septicaemia (aOR 4.36, 95%CI 2.75-6.71).

### Impact of adjusting for age, sex and socioeconomic status

Whilst adjusting for age and sex strongly attenuated associations, as expected, many associations remained (Figure 3 and Tables 2-4) including for cardiovascular (crude OR [cOR] 23.85, 95%CI 23.04-24.70 vs aOR 5.82, 95%CI 5.60-6.05) and eye disorder (cOR 6.10, 95%CI 5.96-6.23 vs aOR 1.42, 95%CI 1.39-1.46) condition groups. However, additional model adjustment for SES did not sizeably change the observed associations for both higher-level conditions groups or individual conditions (Tables 2-4).

## Discussion

This study provides a large-scale population-based characterisation of health conditions in adults with AF. Twenty-two of 23 health condition groups were more prevalent in adults with AF and most (20/23) remained significantly more likely after adjustment for age, sex and SES. This included a broad range of cardiovascular and non-cardiovascular conditions, and also long- and short-term conditions.

Evidence-based guideline recommendations on comorbidity management in AF typically relate to concordant comorbidities whereby management principles of the comorbidity are in keeping with those of AF [8]. For example, beta blockers can lower blood pressure in managing hypertension as well as used as a rate-control strategy for AF. As expected, cardiovascular conditions had the strongest association with AF, likely reflecting structural heart pathology, shared risk factors, and bidirectional causative interaction with conditions such as heart failure [19]. Prevalence estimates for most of our cardiovascular conditions were higher than those reported in population studies of people with incident AF [7, 9, 10], although this trend is expected with comparison with prevalent AF.

Importantly however, a broad range of non-cardiovascular conditions were also highly prevalent and more likely with AF after adjustment for age, sex and SES. This included newly described associations in a population setting between AF and conditions such as hyperparathyroidism and hyperkinetic disorders. Our study therefore provides a resource generalisable to the UK population across diverse conditions for policy makers and researchers to use. Many non-cardiovascular conditions prevalent and more likely in adults with AF in our study have the potential to be discordant whereby management principles are unrelated or potentially conflicting to those of AF. For example, osteoarthritis was highly prevalent in adults with AF (44%) and non-steroidal anti-inflammatory drugs used as analgesia can increase risk of major haemorrhage for AF patients using oral anticoagulants [20]. Similarly, beta-2 agonists used to manage chronic obstructive pulmonary disease (COPD) (prevalence 11% in adults with AF) can antagonise the effects of rate-control strategies for AF. Further research is needed to guide management of discordant comorbidities in patients with AF.

We performed a cross-sectional study with large scale characterisation of adults with AF compared to those without AF. This was done for prevalence estimation, hypothesis generation in quantifying established associations and identifying potentially novel associations, evaluating the impact of risk factors including age, sex, and SES, to support public health planning, policy design, and intervention programs and to help decision makers contextualise populations to other types of evidence. In keeping with the cross-sectional design, associations may not strongly infer causality and may require further study to establish it [16].

We adjusted our analyses for age, sex and SES, to study common risk factors of multimorbidity [21]. Adjustment for risk factors in our analysis frequently attenuated the strength of associations. For example, the unadjusted odds ratio for polymyalgia rheumatica (PMR) reduced from 5.53 (95% CI 5.23-5.85) to 1.22 (95% CI 1.15-1.29) following adjustment for age and sex. This is plausible given AF and PMR have a higher incidence with older age [22]. SES was not a strong risk factor in the relationship between AF and other health conditions with minimal changes in ORs when SES was included. Whilst lower SES has been associated with higher incidence of AF [9] and an increased risk of adverse outcomes and death [23, 24], it suggests that these differences are mediated in another way that should be further explored. We examined the risk factors of age, sex and SES by comparing associations from different logistic regression models. Associations remained in many cases, suggesting that other important risk factors may exist that may benefit from further study using formal causal inference designs with comparative cohorts over time to better understand how comorbidities accrue over time in people with AF.

### Strengths and limitations

Strengths of the study include measuring a broader range of health conditions than previous studies in a large representative sample of the UK population [12], with condition ascertainment from both primary and secondary care. The study therefore provides representative prevalence estimates which are generalisable to the UK population [25]. Our study also has limitations. Diagnoses relied upon clinical coding and we assumed that if a diagnosis was not coded then the person did not have the condition. Whilst this could introduce information bias and potentially underestimate the prevalence of some conditions, the impact was minimised though the use of data linkage between primary and secondary care [25]. The cross-sectional design does not provide information on the temporal relationship between the timing of AF and other health condition diagnosis. Some health conditions will be longstanding and co-existing at the same time as AF whilst others are acute and may be transient. The cross-sectional design may also bias the observed associations between AF presence and health conditions due to different person-years at risk. Longitudinal studies are needed to better understand how comorbidities are acquired over time in people with AF.

### Clinical and policy implications

Our study finding of a wide range of conditions being highly prevalent in adults with AF reinforces placing comorbidity and risk factor management at the forefront of AF management principles. This is in line with the 2024 ESC AF guideline and AF-CARE with the aim to integrate the principles of comorbidity and risk factor management [C], avoidance of stroke and thromboembolism [A], reducing symptoms by rate and rhythm control [R], and evaluation and dynamic reassessment [E], in a framework that includes, among others, shared decision making, including patients and multi-disciplinary teams [8]. Whilst current recommendations typically relate to concordant comorbidities, we report many potential discordant comorbidities which may be important to understand diverse disease-drug and drug-drug interactions. Further research is needed to better inform clinicians of how to optimise management of discordant comorbidities in the context of AF. Furthermore, people with comorbidity reflective of the general population are needed to optimise external validity of clinical trials of emerging AF interventions. Co-existing medical conditions are a common reason for exclusion of participants from clinical trials which may be poorly justified [26]. Our study provides a resource for trialists to compare comorbidities of AF measured in a research population to a general population to support assessment of representativeness.

## Conclusion

Cardiovascular and non-cardiovascular comorbidities are highly prevalent in adults with AF. These may increase the chance of disease-drug interactions and impact on therapeutic choices. Comorbidities should be considered in their entirety for managing individual patients and ensuring representativeness in clinical trials.

## Supplementary Material

Supplementary

## Figures and Tables

**Table 1:** Demographic characteristics of adults with and without atrial fibrillation.

Category	Adults with atrial fibrillation, n (%) (N=34,338)	Adults without atrial fibrillation, n (%) (N=907,739)	Percentage difference (95%CI)
Sex*			
Male	19418 (56.5)	445707 (49.1)	7.4 (6.8 to 8.1)
Female	14919 (43.4)	462026 (50.9)	-7.5 (-8.1 to -6.8)
**Age group**			
18-24	35 (0.1)	81322 (9.0)	-8.9 (-8.9 to -8.8)
25-44	641 (1.9)	290866 (32.0)	-30.1 (-30.4 to -30.0)
45-64	5135 (15.0)	330333 (36.4)	-21.4 (-21.9 to -21.0)
65-84	20532 (59.8)	181956 (20.0)	39.8 (39.2 to 40.3)
85 and over	7995 (23.3)	23262 (26.0)	20.7 (20.2 to 21.2)
**IMD**			
Q1 (least deprived)	8564 (24.9)	230385 (25.4)	-0.5 (-1.0 to 0.1)
Q2	7754 (22.6)	187126 (20.6)	2.0 (1.4 to 2.5)
Q3	7485 (21.8)	182956 (20.2)	1.6 (1.2 to 2.1)
Q4	5948 (17.3)	165136 (18.2)	-0.9 (-1.4 to -0.4)
Q5 (most deprived)	4587 (13.4)	142136 (15.7)	-2.3 (-2.7 to -1.9)

* One adult with atrial fibrillation and six adults without atrial fibrillation had an indeterminate sex; IMD= Index of Multiple Deprivation (in quintiles of the English population).

**Table 2:** Prevalence and odds ratio of health condition groups in adults with atrial fibrillation compared to adults without atrial fibrillation.

Health condition	Adults with AF n (%) (N=34,338)	Adults without AF n (%) (N=907,739)	Unadjusted OR (95%CI)	OR adjusted for age and sex (95%CI)	OR adjusted for age, sex and IMD (95%CI)
Abnormal lipid level	17906 (52.1)	360518 (39.7)	1.65 (1.62-1.69)	0.49 (0.47-0.50)	0.48 (0.47-0.50)
Benign neoplasm	7957 (23.2)	115993 (12.8)	2.06 (2.01-2.11)	1.07 (1.04-1.10)	1.08 (1.05-1.11)
Cardiovascular	30688 (89.4)	236587 (26.1)	23.85 (23.04-24.70)	5.82 (5.60-6.05)	5.81 (5.59-6.03)
Dermatological	16735 (48.7)	387303 (42.7)	1.28 (1.25-1.31)	1.43 (1.40-1.46)	1.44 (1.40-1.47)
Ear nose throat disorder	14770 (43.0)	279142 (30.8)	1.70 (1.66-1.74)	1.23 (1.20-1.26)	1.24 (1.21-1.27)
Endocrine and diabetes	13395 (39.0)	143622 (15.8)	3.40 (3.33-3.48)	1.55 (1.51-1.59)	1.54 (1.50-1.58)
Eye disorder	16494 (48.0)	119496 (13.2)	6.10 (5.96-6.23)	1.42 (1.39-1.46)	1.42 (1.38-1.45)
Gastrointestinal	21246 (61.9)	337617 (37.2)	2.74 (2.68-2.80)	1.34 (1.31-1.38)	1.34 (1.31-1.37)
Genito urinary	14029 (40.9)	151612 (16.7)	3.45 (3.37-3.52)	1.23 (1.20-1.26)	1.23 (1.20-1.26)
Gynaecological	4885 (14.2)	157731 (17.4)	0.79 (0.76-0.81)	0.82 (0.79-0.85)	0.82 (0.79-0.85)
Haematological	9987 (29.1)	103393 (11.4)	3.19 (3.11-3.27)	1.93 (1.88-1.98)	1.92 (1.87-1.97)
Infectious disease	16254 (47.3)	122871 (13.5)	5.74 (5.62-5.87)	3.14 (3.07-3.22)	3.13 (3.06-3.21)
Liver disorder	1149 (3.3)	13067 (1.4)	2.37 (2.23-2.52)	1.48 (1.38-1.57)	1.45 (1.36-1.55)
Mental health	14001 (40.8)	286717 (31.6)	1.49 (1.46-1.52)	1.26 (1.23-1.29)	1.24 (1.21-1.27)
Neurological	7454 (21.7)	135081 (14.9)	1.59 (1.54-1.63)	1.30 (1.27-1.34)	1.30 (1.26-1.33)
Orthopaedic	19856 (57.8)	213466 (23.5)	4.46 (4.36-4.56)	1.32 (1.29-1.35)	1.32 (1.28-1.35)
Primary dermatological malignancy	4183 (12.2)	28496 (3.1)	4.28 (4.13-4.43)	1.09 (1.05-1.14)	1.10 (1.06-1.14)
Primary haematological malignancy	825 (2.4)	5705 (0.6)	3.89 (3.61-4.19)	1.48 (1.37-1.60)	1.48 (1.37-1.60)
Primary solid organ malignancy	5217 (15.2)	41481 (4.6)	3.74 (3.63-3.86)	1.06 (1.03-1.10)	1.07 (1.03-1.10)
Renal	18617 (54.2)	111990 (12.3)	8.41 (8.23-8.60)	2.22 (2.17-2.28)	2.21 (2.16-2.27)
Respiratory	14702 (42.8)	186492 (20.5)	2.90 (2.83-2.96)	2.52 (2.46-2.58)	2.51 (2.45-2.57)
Rheumatological	17325 (50.5)	252637 (27.8)	2.64 (2.58-2.70)	1.03 (1.01-1.06)	1.04 (1.01-1.06)
Secondary malignancy	1261 (3.7)	9791 (1.1)	3.50 (3.29-3.71)	1.06 (1.00-1.13)	1.06 (0.99-1.13)

**Table 3:** Prevalence and odds ratio of the individual cardiovascular conditions in adults with atrial fibrillation compared to adults without atrial fibrillation.

Health condition	Adults with AF, n (%) (N=34,338)	Adults without AF, n (%) (N=907,739)	Unadjusted OR (95%CI)	OR adjusted for age and sex (95%CI)	OR adjusted for age, sex and IMD (95%CI)
Abdominal aortic aneurysm	470 (1.4)	1209 (0.1)	10.41 (9.34-11.57)	2.27 (2.02-2.54)	2.26 (2.01-2.53)
Cardiomyopathy	1552 (4.5)	2591 (0.3)	16.54 (15.51-17.62)	9.58 (8.88-10.35)	9.55 (8.85-10.31)
Conduction disorder	5824 (17.0)	16521 (1.8)	11.02 (10.67-11.38)	2.72 (2.63-2.82)	2.71 (2.62-2.81)
Coronary heart disease	15322 (44.6)	56358 (6.2)	12.17 (11.90-12.45)	3.29 (3.21-3.38)	3.29 (3.21-3.38)
Heart failure	6023 (17.5)	5912 (0.7)	32.45 (31.24-33.70)	9.07 (8.70-9.46)	9.07 (8.70-9.46)
Heart valve disorder	3906 (11.4)	7327 (0.8)	15.77 (15.15-16.42)	5.54 (5.29-5.80)	5.57 (5.32-5.82)
Hypertension	26882 (78.3)	204954 (22.6)	12.36 (12.04-12.69)	2.76 (2.68-2.84)	2.75 (2.67-2.83)
Pericardial effusion noninflammatory	786 (2.3)	1700 (0.2)	12.49 (11.46-13.59)	5.85 (5.31-6.46)	5.83 (5.28-6.42)
Peripheral arterial disease	2336 (6.8)	10233 (1.1)	6.40 (6.11-6.70)	1.97 (1.88-2.07)	1.96 (1.86-2.06)
Sick sinus syndrome	939 (2.7)	728 (0.1)	35.03 (31.78-38.62)	10.94 (9.79-12.23)	10.97 (9.81-12.27)
Stroke	6272 (18.3)	19283 (2.1)	10.30 (9.98-10.62)	2.70 (2.61-2.79)	2.69 (2.60-2.78)
Supraventricular tachycardia	2830 (8.2)	5829 (0.6)	13.90 (13.27-14.55)	8.85 (8.38-9.35)	8.87 (8.40-9.37)
Transient ischaemic attack	3893 (11.3)	12755 (1.4)	8.97 (8.64-9.32)	2.19 (2.10-2.28)	2.18 (2.09-2.27)
Ventricular tachycardia	965 (2.8)	1534 (0.2)	17.08 (15.74-18.52)	7.43 (6.76-8.17)	7.40 (6.73-8.14)

AF= atrial fibrillation; CI= confidence interval; IMD= Index of Multiple Deprivation; OR= odds ratio.

**Table 4:** Prevalence and odds ratio of the twenty most common non-cardiovascular health conditions in adults with atrial fibrillation compared to adults without atrial fibrillation.

Health condition	Adults with AF, n (%) (N=34,338)	Adults without AF, n (%) (N=907,739)	Unadjusted OR (95%CI)	OR adjusted for age and sex (95%CI)	OR adjusted for age, sex and IMD (95%CI)
Osteoarthritis	15120 (44.0)	126637 (14.0)	4.85 (4.75-4.96)	1.12 (1.10-1.15)	1.12 (1.09-1.15)
Chronic kidney disease	13924 (40.5)	70064 (7.7)	8.15 (7.97-8.34)	1.92 (1.87-1.97)	1.91 (1.86-1.96)
Enthesopathies and synovial disorders	12801 (37.3)	207303 (22.8)	2.01 (1.96-2.05)	0.85 (0.83-0.87)	0.85 (0.83-0.87)
Cataract	11649 (33.9)	63595 (7.0)	6.82 (6.65-6.98)	1.29 (1.25-1.32)	1.29 (1.25-1.32)
Other infection	10945 (31.9)	69300 (7.6)	5.66 (5.53-5.80)	3.18 (3.10-3.26)	3.16 (3.08-3.24)
Dermatitis (atopic/ contact/ other/ unspecified)	10629 (31.0)	235761 (26.0)	1.28 (1.25-1.31)	1.34 (1.31-1.38)	1.35 (1.31-1.38)
Raised cholesterol	10515 (30.6)	280223 (30.9)	0.99 (0.97-1.01)	0.36 (0.35-0.37)	0.36 (0.35-0.37)
Raised LDL (low-density lipoprotein) cholesterol	9912 (28.9)	239899 (26.4)	1.13 (1.10-1.16)	0.44 (0.43-0.46)	0.45 (0.43-0.46)
Bacterial diseases (excluding tuberculosis)	9139 (26.6)	53800 (5.9)	5.76 (5.61-5.90)	2.64 (2.57-2.71)	2.62 (2.55-2.70)
Urinary tract infections	8453 (24.6)	40778 (4.5)	6.94 (6.76-7.13)	2.57 (2.50-2.65)	2.56 (2.49-2.63)
Acute kidney injury	8159 (23.8)	25371 (2.8)	10.84 (10.54-11.14)	2.76 (2.68-2.85)	2.76 (2.68-2.85)
Gastro oesophageal reflux disease, oesophagitis and oesophageal ulcer	8102 (23.6)	109975 (12.1)	2.24 (2.18-2.30)	1.08 (1.05-1.11)	1.07 (1.04-1.10)
Hearing loss	8079 (23.5)	82363 (9.1)	3.08 (3.00-3.16)	1.33 (1.30-1.37)	1.33 (1.29-1.37)
Lower respiratory tract infections	7559 (22.0)	32827 (3.6)	7.52 (7.32-7.73)	2.84 (2.76-2.93)	2.84 (2.75-2.93)
Depression	7373 (21.5)	187762 (20.7)	1.05 (1.02-1.08)	0.95 (0.92-0.98)	0.93 (0.91-0.96)
Abdominal hernia	6437 (18.7)	67596 (7.4)	2.87 (2.79-2.95)	1.27 (1.23-1.31)	1.27 (1.23-1.31)
Hyperplasia of prostate	6195 (18.0)	32634 (3.6)	5.90 (5.73-6.08)	1.16 (1.12-1.21)	1.17 (1.12-1.21)
Type 2 diabetes mellitus	6629 (19.3)	51293 (5.7)	3.99 (3.88-4.11)	1.25 (1.21-1.28)	1.23 (1.20-1.27)
Anxiety disorders	5833 (17.0)	141632 (15.6)	1.11 (1.08-1.14)	1.02 (0.99-1.06)	1.01 (0.98-1.04)
Low HDL (high-density lipoprotein) cholesterol	5820 (16.9)	71571 (7.9)	2.38 (2.32-2.45)	1.33 (1.29-1.38)	1.32 (1.28-1.36)

AF= atrial fibrillation; CI= confidence interval; IMD= Index of Multiple Deprivation; OR= odds ratio.

## Data Availability

Deidentified patient data used in this study are not publicly available and may be obtained from the Clinical Practice Research Datalink according to their standard terms and conditions (https://www.cprd.com/).

## What is already known on this topic

The AF-CARE framework places comorbidities and risk factors as the initial and central component of AF management. However, comorbidity is often poorly or incompletely characterised in AF patients.

## What this study adds

Cardiovascular conditions were highly prevalent in adults with AF and strongly associated with the presence of AF even after accounting for age and sex. However, the same was true for a very wide range of non-cardiovascular conditions.

## How this study might affect research, practice or policy

Comorbidities should be considered in their entirety to manage the scope of potential disease-drug and drug-drug interactions when considering treatment options for AF.

As the prevalence of AF increases, anticipating the impact of comorbidity will be important for future healthcare planning.
