# Supplementary material for: Health conditions in adults with atrial fibrillation compared with the general population: a population-based cross-sectional analysis
Source: Heart. Author manuscript; Available in PMC 2025 Mar 31. (PMC7617536; doi:10.1136/heartjnl-2024-324618)
Supplement: Supplementary [file EMS203667-supplement-Supplementary.pdf]

Health conditions in adults with atrial fibrillation compared with the general population: a population-based cross-sectional analysis.

SUPPLEMENTARY MATERIAL

1. Thomas J. Downes, Division of Population Health and Genomics, University of Dundee, Dundee, UK.
2. Bruce Guthrie, Advanced Care Research Centre, Usher Institute, University of Edinburgh, Edinburgh, UK.
3. David Moreno-Martos, Division of Population Health and Genomics, University of Dundee, Dundee, UK.
4. Daniel R. Morales, Division of Population Health and Genomics, University of Dundee, Dundee, UK.

Supplementary table 1 Primary care codes to identify adults with atrial fibrillation.

Medcode	Read code (v2)	Description
90190	9Os3.00	Atrial fibrillation monitoring verbal invite
1664	G573000	Atrial fibrillation
93460	14AR.00	History of atrial flutter
28994	212R.00	Atrial fibrillation resolved
90187	9Os0.00	Atrial fibrillation monitoring first letter
96277	G573400	Permanent atrial fibrillation
1757	G573100	Atrial flutter
57832	9Os..00	Atrial fibrillation monitoring administration
3757	3272.00	ECG: atrial fibrillation
35127	G573300	Non-rheumatic atrial fibrillation
90191	9Os4.00	Atrial fibrillation monitoring telephone invite
39114	9hF1.00	Excepted from atrial fibrillation qual indic: Inform dissent
2212	G573.00	Atrial fibrillation and flutter
45773	6A9..00	Atrial fibrillation annual review
63350	9hF..00	Exception reporting: atrial fibrillation quality indicators
90189	9Os2.00	Atrial fibrillation monitoring third letter
90188	9Os1.00	Atrial fibrillation monitoring second letter
6345	14AN.00	H/O: atrial fibrillation
6771	3273.00	ECG: atrial flutter
18746	662S.00	Atrial fibrillation monitoring
96076	G573500	Persistent atrial fibrillation
23437	G573z00	Atrial fibrillation and flutter NOS
1268	G573200	Paroxysmal atrial fibrillation

Supplementary table 2 ICD-10 codes related to atrial fibrillation.

ICD-10 code	ICD-10 term
I48.0	Paroxysmal atrial fibrillation
I48.1	Persistent atrial fibrillation
I48.2	Chronic atrial fibrillation
I48.3	Typical atrial flutter
I48.4	Atypical atrial flutter
I48.9	Atrial fibrillation and atrial flutter, unspecified

Supplementary table 3 List of codelists excluded from analysis and reason for exclusion.

Codelists excluded	Reason for excluding
Diabetes (aggregate)	Type 1 and 2 diabetes mellitus, diabetic neuropathy and diabetic ophthalmic complications as individual conditions only
Postcoital and contact bleeding	Symptoms rather than a disease
Respiratory failure	Non-specific term
Sickle-cell trait	Not a disease but a carrier of one gene responsible for sickle cell disease
Thalassaemia trait	Not a disease but a carrier of one gene responsible for thalassaemia
Pleural plaque	Asymptomatic and not a disease in itself
Female infertility	Risk of double coding for a male and female partner
Male infertility	Risk of double coding for a male and female partner
Patent ductus arteriosus	Perinatal condition
Bacterial sepsis of newborn	Perinatal condition
Cerebral palsy	Perinatal condition
Congenital malformations of cardiac septa	Perinatal condition
Diaphragmatic hernia	Perinatal condition
High birth weight	Perinatal condition
Intrauterine hypoxia	Perinatal condition
Neonatal jaundice (excluding haemolytic disease of the newborn)	Perinatal condition
Post-term infant	Perinatal condition
Prematurity	Perinatal condition
Respiratory distress of newborn	Perinatal condition
Slow foetal growth or low birth weight	Perinatal condition
Spina bifida	Perinatal condition
Undescended testicle	Perinatal condition

Supplementary table 4 Aggregate of individual codelists.

Aggregate	Codelists
Cardiomyopathy	Dilated cardiomyopathy<br>Hypertrophic cardiomyopathy<br>Other cardiomyopathy
Conduction disorder	Atrioventricular block first degree<br>Atrioventricular block second degree<br>Atrioventricular block complete<br>Bifascicular block<br>Left bundle branch block<br>Right bundle branch block<br>Trifascicular block
Coronary heart disease	Coronary heart disease not otherwise specified<br>Myocardial infarction<br>Stable angina<br>Unstable angina
Fracture of hip, wrist or vertebra	Collapsed vertebra<br>Fracture of hip<br>Fracture of wrist
Gastritis duodenitis and peptic ulcer disease	Gastritis and duodenitis<br>Peptic ulcer disease
Gastro oesophageal reflux disease oesophagitis and oesophageal ulcer	Gastro oesophageal reflux disease<br>Oesophagitis and oesophageal ulcer
Heart valve disorder	Multiple valve disease<br>Nonrheumatic aortic valve disorders<br>Nonrheumatic mitral valve disorders<br>Rheumatic valve disease
Inflammatory bowel disease	Crohn’s disease<br>Ulcerative colitis
Nervous system infection	Encephalitis<br>Meningitis<br>Other nervous system infections
Osteoarthritis	Osteoarthritis (excluding spine)<br>Spondylosis
Other infection	Other or unspecified infectious organisms<br>Infections of other or unspecified organs
Peripheral or autonomic neuropathy (excluding cranial nerve or carpal tunnel syndrome)	Diabetic neurological complications<br>Disorders of autonomic nervous system<br>Peripheral neuropathies (excluding cranial nerve and carpal tunnel syndromes)
Primary malignancy other	Primary malignancy multiple independent sites<br>Primary malignancy other organs
Pulmonary hypertension	Primary pulmonary hypertension<br>Secondary pulmonary hypertension
Severe liver disease	Liver failure<br>Liver fibrosis sclerosis and cirrhosis<br>Oesophageal varices<br>Portal hypertension
Stroke	Intracerebral haemorrhage<br>Ischaemic stroke<br>Stroke NOS
Thrombocytopenia	Primary or idiopathic thrombocytopaenia<br>Secondary or other thrombocytopaenia

Supplementary table 5 Proportion of adults with a previous lipid blood test.

	With AF, n (%) (N= 34,338)	Without AF, n (%) (N= 907,739)
Raised cholesterol	32750 (95.4)	534480 (58.9)
Low high-density lipoprotein (HDL) cholesterol	31534 (91.8)	505534 (55.7)
Raised low-density lipoprotein (LDL) cholesterol	30181 (87.9)	460159 (50.7)
Raised triglycerides	30823 (89.8)	466995 (51.4)

AF= atrial fibrillation.

Supplementary table 6 Prevalence and odds ratio of endocrine and lipid conditions in adults with atrial fibrillation compared to adults without atrial fibrillation.

Health condition	Adults with AF, n (%) (N=34,338)	Adults without AF, n (%) (N=907,739)	Unadjusted OR (95%CI)	OR adjusted for age and sex (95%CI)	OR adjusted for age, sex and IMD (95%CI)
**Endocrine and diabetes**					
Hyperparathyroidism	421 (1.2)	2510 (0.3)	4.48 (4.03-4.96)	1.62 (1.45-1.81)	1.61 (1.44-1.80)
Obesity	4070 (11.9)	55054 (6.1)	2.08 (2.01-2.15)	1.52 (1.46-1.57)	1.49 (1.44-1.54)
Syndrome of inappropriate secretion of antidiuretic hormone	172 (0.5)	694 (0.1)	6.58 (5.55-7.76)	1.53 (1.28-1.82)	1.53 (1.28-1.82)
Type 1 diabetes mellitus	131 (0.4)	3804 (0.4)	0.91 (0.76-1.08)	1.17 (0.97-1.40)	1.17 (0.97-1.39)
Type 2 diabetes mellitus	6629 (19.3)	51293 (5.7)	3.99 (3.88-4.11)	1.25 (1.21-1.28)	1.23 (1.20-1.27)
Thyroid disease	5606 (16.3)	54860 (6.0)	3.03 (2.94-3.13)	1.53 (1.48-1.58)	1.52 (1.47-1.58)
**Abnormal lipid level**					
Low HDL (high-density lipoprotein) cholesterol	5820 (16.9)	71571 (7.9)	2.38 (2.32-2.45)	1.33 (1.29-1.38)	1.32 (1.28-1.36)
Raised cholesterol	10515 (30.6)	280223 (30.9)	0.99 (0.97-1.01)	0.36 (0.35-0.37)	0.36 (0.35-0.37)
Raised LDL (low-density lipoprotein) cholesterol	9912 (28.9)	239899 (26.4)	1.13 (1.10-1.16)	0.44 (0.43-0.46)	0.45 (0.43-0.46)
Raised triglycerides	3542 (10.3)	61879 (6.8)	1.57 (1.52-1.63)	0.79 (0.76-0.82)	0.78 (0.75-0.81)

CI= confidence interval; IMD= Index of Multiple Deprivation; OR= odds ratio.

Supplementary table 7 Prevalence and odds ratio of mental health and neurological conditions in adults with atrial fibrillation compared to adults without atrial fibrillation.

Health condition	Adults with AF, n (%) (N=34,338)	Adults without AF, n (%) (N=907,739)	Unadjusted OR (95%CI)	OR adjusted for age and sex (95%CI)	OR adjusted for age, sex and IMD (95%CI)
**Mental health**					
Alcohol problems	2315 (6.7)	35086 (3.9)	1.80 (1.72-1.88)	1.38 (1.32-1.45)	1.36 (1.29-1.42)
Anxiety disorders	5833 (17.0)	141632 (15.6)	1.11 (1.08-1.14)	1.02 (0.99-1.06)	1.01 (0.98-1.04)
Autism and Asperger's syndrome	32 (0.1)	3055 (0.3)	0.28 (0.19-0.38)	2.56 (1.76-3.60)	2.54 (1.74-3.58)
Bipolar affective disorder and mania	126 (0.4)	3543 (0.4)	0.94 (0.78-1.12)	0.79 (0.66-0.95)	0.78 (0.64-0.93)
Delirium (not induced by alcohol and other psychoactive substances)	275 (0.8)	894 (0.1)	8.19 (7.14-9.36)	2.28 (1.97-2.63)	2.26 (1.95-2.61)
Dementia	3802 (11.1)	12881 (1.4)	8.65 (8.33-8.98)	1.38 (1.32-1.43)	1.37 (1.32-1.43)
Depression	7373 (21.5)	187762 (20.7)	1.05 (1.02-1.08)	0.95 (0.92-0.98)	0.93 (0.91-0.96)
Eating disorders	35 (0.1)	3697 (0.4)	0.25 (0.18-0.34)	0.67 (0.47-0.92)	0.68 (0.47-0.93)
Hyperkinetic disorders	11 (0.0)	2617 (0.3)	0.11 (0.06-0.19)	2.14 (1.10-3.71)	2.12 (1.09-3.67)
Intellectual disability	179 (0.5)	6542 (0.7)	0.72 (0.62-0.83)	1.40 (1.19-1.63)	1.35 (1.15-1.57)
Obsessive compulsive disorder	113 (0.3)	4981 (0.5)	0.60 (0.49-0.72)	0.95 (0.78-1.14)	0.94 (0.77-1.14)
Personality disorders	212 (0.6)	5722 (0.6)	0.98 (0.85-1.12)	1.03 (0.89-1.19)	1.00 (0.86-1.15)
Schizophrenia, schizotypal and delusional disorders	242 (0.7)	6455 (0.7)	0.99 (0.87-1.12)	0.84 (0.73-0.95)	0.81 (0.70-0.92)
Substance misuse	374 (1.1)	14891 (1.6)	0.66 (0.59-0.73)	1.01 (0.90-1.12)	0.96 (0.86-1.07)
**Neurological**					
Bell's palsy	594 (1.7)	8300 (0.9)	1.91 (1.75-2.07)	1.20 (1.09-1.30)	1.19 (1.08-1.29)
Epilepsy	807 (2.4)	14704 (1.6)	1.46 (1.36-1.57)	1.43 (1.32-1.54)	1.40 (1.30-1.51)
Essential tremor	395 (1.2)	2820 (0.3)	3.73 (3.35-4.15)	1.42 (1.26-1.58)	1.42 (1.26-1.58)
Intracranial hypertension	15 (0.0)	725 (0.1)	0.55 (0.31-0.88)	1.85 (1.04-3.04)	1.78 (1.00-2.93)
Migraine	2468 (7.2)	81173 (8.9)	0.79 (0.76-0.82)	0.99 (0.95-1.03)	0.99 (0.95-1.03)
Motor neurone disease	42 (0.1)	327 (0.0)	3.40 (2.43-4.63)	1.24 (0.88-1.73)	1.24 (0.87-1.72)
Multiple sclerosis	123 (0.4)	3080 (0.3)	1.06 (0.88-1.26)	0.76 (0.63-0.91)	0.76 (0.63-0.92)
Myasthenia gravis	52 (0.2)	431 (0.0)	3.19 (2.37-4.21)	1.36 (0.99-1.82)	1.36 (0.99-1.83)
Parkinson's disease	656 (1.9)	3378 (0.4)	5.21 (4.79-5.67)	1.13 (1.03-1.23)	1.13 (1.03-1.23)
Peripheral or autonomic neuropathy (excluding cranial nerve or carpal tunnel syndrome)	2506 (7.3)	23478 (2.6)	2.97 (2.84-3.09)	1.30 (1.24-1.36)	1.29 (1.23-1.35)
Subarachnoid haemorrhage	165 (0.5)	1547 (0.2)	2.83 (2.40-3.31)	1.28 (1.07-1.51)	1.27 (1.07-1.50)
Subdural haematoma (non-traumatic)	307 (0.9)	912 (0.1)	8.97 (7.87-10.20)	2.28 (1.99-2.62)	2.28 (1.98-2.61)
Trigeminal neuralgia	501 (1.5)	6137 (0.7)	2.18 (1.98-2.38)	0.99 (0.90-1.09)	0.99 (0.90-1.09)

CI= confidence interval; IMD= Index of Multiple Deprivation; OR= odds ratio.

Supplementary table 8 Prevalence and odds ratio of respiratory, gastrointestinal and liver conditions in adults with atrial fibrillation compared to adults without atrial fibrillation.

Health condition	Adults with AF, n (%) (N=34,338)	Adults without AF, n (%) (N=907,739)	Unadjusted OR (95%CI)	OR adjusted for age and sex (95%CI)	OR adjusted for age, sex and IMD (95%CI)
**Respiratory**					
Asbestosis	271 (0.8)	959 (0.1)	7.52 (6.56-8.60)	1.47 (1.28-1.70)	1.47 (1.27-1.69)
Asthma	5037 (14.7)	136691 (15.1)	0.97 (0.94-1.00)	1.42 (1.37-1.46)	1.41 (1.37-1.46)
Bronchiectasis	1109 (3.2)	5880 (0.6)	5.12 (4.79-5.46)	1.60 (1.49-1.71)	1.60 (1.49-1.71)
Chronic Obstructive Pulmonary Disease	3703 (10.8)	22013 (2.4)	4.86 (4.69-5.04)	1.39 (1.33-1.44)	1.37 (1.32-1.43)
Cystic fibrosis	13 (0.0)	420 (0.0)	0.82 (0.45-1.36)	0.93 (0.50-1.58)	0.92 (0.50-1.57)
Other interstitial pulmonary diseases with fibrosis	665 (1.9)	2683 (0.3)	6.66 (6.11-7.25)	1.60 (1.46-1.75)	1.59 (1.45-1.74)
Pleural effusion	5662 (16.5)	15963 (1.8)	11.03 (10.68-11.39)	3.55 (3.42-3.67)	3.53 (3.41-3.66)
Pneumothorax	250 (0.7)	3761 (0.4)	1.76 (1.55-2.00)	1.30 (1.13-1.49)	1.30 (1.13-1.48)
Pulmonary collapse (excluding pneumothorax)	1738 (5.1)	8254 (0.9)	5.81 (5.51-6.12)	2.12 (2.01-2.25)	2.10 (1.99-2.23)
Pulmonary embolism	1353 (3.9)	6378 (0.7)	5.80 (5.46-6.15)	2.24 (2.10-2.39)	2.23 (2.09-2.37)
Pulmonary hypertension	1648 (4.8)	1811 (0.2)	25.22 (23.57-26.98)	8.61 (7.97-9.30)	8.57 (7.93-9.26)
Sarcoidosis	129 (0.4)	1935 (0.2)	1.77 (1.47-2.10)	0.88 (0.73-1.06)	0.89 (0.74-1.07)
Sleep apnoea	1358 (4.0)	12132 (1.3)	3.04 (2.87-3.22)	1.89 (1.78-2.01)	1.88 (1.77-2.00)
**Gastrointestinal**					
Abdominal hernia	6437 (18.7)	67596 (7.4)	2.87 (2.79-2.95)	1.27 (1.23-1.31)	1.27 (1.23-1.31)
Anal fissure	1058 (3.1)	26907 (3.0)	1.04 (0.98-1.11)	1.11 (1.04-1.18)	1.12 (1.05-1.20)
Angiodysplasia of colon	222 (0.6)	915 (0.1)	6.45 (5.55-7.45)	2.01 (1.71-2.35)	2.00 (1.70-2.33)
Anorectal fistula	382 (1.1)	5909 (0.7)	1.72 (1.54-1.90)	1.31 (1.18-1.46)	1.31 (1.17-1.46)
Anorectal prolapse	384 (1.1)	3581 (0.4)	2.86 (2.56-3.17)	1.20 (1.07-1.34)	1.20 (1.08-1.34)
Appendicitis	3766 (11)	64683 (7.1)	1.61 (1.55-1.66)	1.04 (1.00-1.07)	1.04 (1.00-1.08)
Barrett's oesophagus	886 (2.6)	7324 (0.8)	3.26 (3.03-3.49)	1.06 (0.99-1.15)	1.06 (0.99-1.14)
Cholangitis	546 (1.6)	2287 (0.3)	6.40 (5.82-7.02)	2.10 (1.89-2.32)	2.08 (1.88-2.30)
Cholecystitis	1742 (5.1)	19378 (2.1)	2.45 (2.33-2.58)	1.46 (1.39-1.54)	1.45 (1.37-1.53)
Cholelithiasis	2125 (6.2)	24328 (2.7)	2.40 (2.29-2.51)	1.26 (1.20-1.32)	1.25 (1.19-1.31)
Coeliac disease	219 (0.6)	4134 (0.5)	1.40 (1.22-1.60)	1.13 (0.98-1.30)	1.13 (0.98-1.30)
Diverticular disease of intestine (acute and chronic)	4716 (13.7)	28765 (3.2)	4.86 (4.71-5.03)	1.23 (1.19-1.27)	1.23 (1.19-1.28)
Gastritis duodenitis and peptic ulcer disease	4780 (13.9)	56071 (6.2)	2.46 (2.38-2.54)	1.26 (1.22-1.31)	1.25 (1.21-1.29)
Gastro oesophageal reflux disease, oesophagitis and oesophageal ulcer	8102 (23.6)	109975 (12.1)	2.24 (2.18-2.30)	1.08 (1.05-1.11)	1.07 (1.04-1.10)
Inflammatory bowel disease	486 (1.4)	8825 (1.0)	1.46 (1.33-1.60)	1.03 (0.94-1.14)	1.04 (0.94-1.14)
Irritable bowel syndrome	2587 (7.5)	68710 (7.6)	0.99 (0.95-1.04)	0.91 (0.87-0.95)	0.92 (0.88-0.96)
Pancreatitis	645 (1.9)	5416 (0.6)	3.19 (2.93-3.46)	1.62 (1.48-1.77)	1.60 (1.47-1.75)
Pilonidal cyst sinus	442 (1.3)	11569 (1.3)	1.01 (0.92-1.11)	1.19 (1.08-1.31)	1.19 (1.07-1.31)
Volvulus	195 (0.6)	1312 (0.1)	3.95 (3.38-4.58)	1.57 (1.33-1.84)	1.56 (1.33-1.83)
**Liver disorders**					
Alcoholic liver disease	154 (0.4)	1615 (0.2)	2.53 (2.13-2.97)	1.42 (1.18-1.68)	1.39 (1.16-1.65)
Autoimmune liver disease	73 (0.2)	763 (0.1)	2.53 (1.97-3.20)	1.28 (0.98-1.63)	1.27 (0.98-1.63)
Chronic viral hepatitis	43 (0.1)	1808 (0.2)	0.63 (0.46-0.84)	0.59 (0.43-0.80)	0.58 (0.42-0.78)
Fatty liver	644 (1.9)	7536 (0.8)	2.28 (2.10-2.47)	1.43 (1.32-1.56)	1.42 (1.30-1.54)
Severe liver disease	556 (1.6)	4482 (0.5)	3.32 (3.03-3.62)	1.79 (1.63-1.97)	1.76 (1.60-1.93)

CI= confidence interval; IMD= Index of Multiple Deprivation; OR= odds ratio.

Supplementary table 9 Prevalence and odds ratio of orthopaedic and rheumatological conditions in adults with atrial fibrillation compared to adults without atrial fibrillation.

Health condition	Adults with AF, n (%) (N=34,338)	Adults without AF, n (%) (N=907,739)	Unadjusted OR (95%CI)	OR adjusted for age and sex (95%CI)	OR adjusted for age, sex and IMD (95%CI)
**Orthopaedic**					
Carpal tunnel syndrome	2986 (8.7)	43020 (4.7)	1.91 (1.84-1.99)	0.95 (0.91-0.99)	0.95 (0.91-0.99)
Fracture of hip, wrist or vertebra	4990 (14.5)	45538 (5.0)	3.22 (3.12-3.32)	1.64 (1.58-1.69)	1.63 (1.58-1.69)
Intervertebral disc disorders	1654 (4.8)	24456 (2.7)	1.83 (1.74-1.92)	0.90 (0.86-0.95)	0.91 (0.86-0.96)
Osteoarthritis	15120 (44.0)	126637 (14.0)	4.85 (4.75-4.96)	1.12 (1.10-1.15)	1.12 (1.09-1.15)
Osteoporosis	3505 (10.2)	23274 (2.6)	4.32 (4.16-4.48)	1.11 (1.06-1.15)	1.11 (1.06-1.15)
Scoliosis	348 (1.0)	6045 (0.7)	1.53 (1.37-1.70)	1.38 (1.23-1.55)	1.39 (1.24-1.56)
Spinal stenosis	1362 (4.0)	10513 (1.2)	3.52 (3.33-3.73)	1.13 (1.07-1.20)	1.13 (1.06-1.20)
Spondylolisthesis	494 (1.4)	4764 (0.5)	2.77 (2.52-3.03)	1.03 (0.93-1.13)	1.03 (0.93-1.13)
**Rheumatological**					
Ankylosing spondylitis	174 (0.5)	2291 (0.3)	2.01 (1.72-2.34)	1.09 (0.92-1.27)	1.08 (0.92-1.27)
Enteropathic arthropathy	5 (0.0)	143 (0.0)	0.92 (0.33-2.03)	0.95 (0.33-2.16)	0.93 (0.32-2.13)
Enthesopathies synovial disorders	12801 (37.3)	207303 (22.8)	2.01 (1.96-2.05)	0.85 (0.83-0.87)	0.85 (0.83-0.87)
Giant cell arteritis	329 (1.0)	1641 (0.2)	5.34 (4.74-6.01)	1.33 (1.17-1.51)	1.33 (1.17-1.50)
Gout	4734 (13.8)	28155 (3.1)	5.00 (4.83-5.16)	1.65 (1.59-1.71)	1.65 (1.59-1.71)
Juvenile arthritis	5 (0.0)	541 (0.1)	0.24 (0.09-0.53)	0.76 (0.27-1.67)	0.76 (0.27-1.68)
Lupus erythematosus local and systemic	86 (0.3)	1433 (0.2)	1.59 (1.27-1.96)	1.08 (0.86-1.35)	1.07 (0.85-1.34)
Polymyalgia rheumatica	1572 (4.6)	7802 (0.9)	5.53 (5.23-5.85)	1.22 (1.15-1.29)	1.22 (1.15-1.29)
Post-infective and reactive arthropathies	52 (0.2)	1089 (0.1)	1.26 (0.94-1.65)	0.98 (0.73-1.30)	1.00 (0.74-1.32)
Post-viral fatigue syndrome, neurasthenia and fibromyalgia	733 (2.1)	17949 (2.0)	1.08 (1.00-1.16)	0.87 (0.80-0.94)	0.85 (0.79-0.92)
Psoriatic arthropathy	150 (0.4)	3047 (0.3)	1.30 (1.10-1.53)	0.79 (0.67-0.94)	0.79 (0.67-0.94)
Raynaud's syndrome	765 (2.2)	11810 (1.3)	1.73 (1.60-1.86)	1.29 (1.19-1.39)	1.29 (1.19-1.39)
Rheumatoid arthritis	830 (2.4)	7741 (0.9)	2.88 (2.68-3.09)	1.25 (1.16-1.35)	1.24 (1.15-1.34)
Sjogren's disease	112 (0.3)	1251 (0.1)	2.37 (1.94-2.86)	1.08 (0.88-1.31)	1.08 (0.88-1.31)
Systemic sclerosis	26 (0.1)	352 (0.0)	1.95 (1.28-2.85)	1.02 (0.66-1.52)	1.02 (0.66-1.52)

CI= confidence interval; IMD= Index of Multiple Deprivation; OR= odds ratio.

Supplementary table 10 Prevalence and odds ratio of genitourinary, gynaecological and renal conditions in adults with atrial fibrillation compared to adults without atrial fibrillation.

Health condition	Adults with AF, n (%) (N=34,338)	Adults without AF, n (%) (N=907,739)	Unadjusted OR (95%CI)	OR adjusted for age and sex (95%CI)	OR adjusted for age, sex and IMD (95%CI)
**Genitourinary disorder**					
Erectile dysfunction	4890 (14.2)	48404 (5.3)	2.95 (2.86-3.04)	1.01 (0.97-1.05)	1.01 (0.97-1.04)
Hydrocoele (including infected)	760 (2.2)	9146 (1.0)	2.22 (2.06-2.39)	1.56 (1.44-1.69)	1.56 (1.44-1.69)
Hyperplasia of prostate	6195 (18.0)	32634 (3.6)	5.90 (5.73-6.08)	1.16 (1.12-1.21)	1.17 (1.12-1.21)
Neuromuscular dysfunction of bladder	848 (2.5)	9462 (1.0)	2.40 (2.24-2.58)	1.12 (1.04-1.20)	1.11 (1.03-1.20)
Non acute cystitis	285 (0.8)	2527 (0.3)	3.00 (2.65-3.38)	1.27 (1.11-1.44)	1.27 (1.11-1.44)
Obstructive and reflux uropathy	993 (2.9)	9877 (1.1)	2.71 (2.53-2.89)	1.47 (1.37-1.58)	1.46 (1.36-1.57)
Urinary incontinence	3224 (9.4)	40822 (4.5)	2.20 (2.12-2.28)	1.19 (1.14-1.24)	1.18 (1.13-1.23)
Urolithiasis	2408 (7.0)	34905 (3.8)	1.89 (1.81-1.97)	0.96 (0.92-1.01)	0.96 (0.92-1.00)
**Gynaecological**					
Cervical intraepithelial neoplasia	129 (0.4)	9264 (1.0)	0.37 (0.31-0.43)	0.56 (0.47-0.67)	0.55 (0.46-0.66)
Dysmenorrhoea	280 (0.8)	36114 (4.0)	0.20 (0.18-0.22)	0.57 (0.50-0.64)	0.56 (0.50-0.63)
Endometrial hyperplasia and hypertrophy	209 (0.6)	5037 (0.6)	1.10 (0.95-1.26)	0.81 (0.70-0.94)	0.81 (0.70-0.93)
Endometriosis	162 (0.5)	9890 (1.1)	0.43 (0.37-0.50)	0.58 (0.49-0.68)	0.59 (0.50-0.69)
Female genital prolapse	2090 (6.1)	25545 (2.8)	2.24 (2.14-2.34)	0.86 (0.82-0.90)	0.86 (0.82-0.91)
Female pelvic inflammatory disease	58 (0.2)	2654 (0.3)	0.58 (0.44-0.74)	0.61 (0.47-0.79)	0.60 (0.46-0.79)
Menorrhagia and polymenorrhoea	1378 (4.0)	87273 (9.6)	0.39 (0.37-0.41)	0.51 (0.48-0.54)	0.50 (0.47-0.53)
Polycystic ovarian syndrome	30 (0.1)	7090 (0.8)	0.11 (0.08-0.16)	0.77 (0.52-1.09)	0.77 (0.52-1.08)
Postmenopausal bleeding	1733 (5.0)	22151 (2.4)	2.12 (2.02-2.23)	0.82 (0.77-0.86)	0.82 (0.78-0.86)
**Renal**					
Acute kidney injury	8159 (23.8)	25371 (2.8)	10.84 (10.54-11.14)	2.76 (2.68-2.85)	2.76 (2.68-2.85)
Chronic kidney disease	13924 (40.5)	70064 (7.7)	8.15 (7.97-8.34)	1.92 (1.87-1.97)	1.91 (1.86-1.96)
End stage renal disease	4261 (12.4)	32708 (3.6)	3.79 (3.66-3.92)	1.26 (1.21-1.30)	1.25 (1.21-1.30)
Glomerulonephritis	787 (2.3)	4461 (0.5)	4.75 (4.40-5.12)	2.16 (1.99-2.35)	2.15 (1.98-2.33)
Tubulointerstitial nephritis	277 (0.8)	4577 (0.5)	1.60 (1.42-1.81)	2.57 (2.25-2.92)	2.51 (2.19-2.85)

CI= confidence interval; IMD= Index of Multiple Deprivation; OR= odds ratio.

Supplementary table 11 Prevalence and odds ratio of ear, nose and throat conditions, eye conditions, dermatological conditions and Down's syndrome in adults with atrial fibrillation compared to adults without atrial fibrillation.

Health condition	Adults with AF, n (%) (N=34,338)	Adults without AF, n (%) (N=907,739)	Unadjusted OR (95%CI)	OR adjusted for age and sex (95%CI)	OR adjusted for age, sex and IMD (95%CI)
**Ear nose and throat disorder**					
Allergic and chronic rhinitis	5701 (16.6)	159976 (17.6)	0.93 (0.90-0.96)	1.10 (1.07-1.14)	1.11 (1.08-1.14)
Chronic sinusitis	2844 (8.3)	61251 (6.7)	1.25 (1.20-1.30)	0.83 (0.79-0.86)	0.83 (0.80-0.87)
Hearing loss	8079 (23.5)	82363 (9.1)	3.08 (3.00-3.16)	1.33 (1.30-1.37)	1.33 (1.29-1.37)
Hypertrophy of nasal turbinates	113 (0.3)	4700 (0.5)	0.63 (0.52-0.76)	0.82 (0.67-0.99)	0.81 (0.67-0.98)
Meniere's disease	420 (1.2)	3651 (0.4)	3.07 (2.77-3.39)	1.09 (0.98-1.21)	1.09 (0.97-1.21)
Tinnitus	2792 (8.1)	36126 (4.0)	2.14 (2.05-2.22)	0.97 (0.93-1.01)	0.97 (0.93-1.01)
Eye disorder					
Anterior and intermediate uveitis	527 (1.5)	7348 (0.8)	1.91 (1.75-2.09)	1.00 (0.91-1.09)	1.00 (0.91-1.10)
Blindness	1274 (3.7)	7746 (0.9)	4.48 (4.21-4.75)	1.54 (1.44-1.64)	1.53 (1.43-1.63)
Cataract	11649 (33.9)	63595 (7.0)	6.82 (6.65-6.98)	1.29 (1.25-1.32)	1.29 (1.25-1.32)
Diabetic ophthalmic complications	2777 (8.1)	18902 (2.1)	4.14 (3.97-4.31)	1.40 (1.34-1.46)	1.39 (1.33-1.45)
Eye infections	84 (0.2)	614 (0.1)	3.62 (2.86-4.52)	2.12 (1.65-2.70)	2.10 (1.63-2.67)
Glaucoma	3863 (11.2)	25352 (2.8)	4.41 (4.26-4.57)	0.98 (0.94-1.02)	0.98 (0.94-1.02)
Keratitis	335 (1.0)	5152 (0.6)	1.73 (1.54-1.93)	1.14 (1.02-1.28)	1.15 (1.02-1.29)
Macular degeneration	2920 (8.5)	13645 (1.5)	6.09 (5.84-6.35)	1.17 (1.12-1.22)	1.17 (1.12-1.22)
Posterior uveitis	41 (0.1)	604 (0.1)	1.80 (1.29-2.43)	1.00 (0.71-1.37)	1.00 (0.71-1.37)
Ptosis of eyelid	906 (2.6)	6612 (0.7)	3.69 (3.44-3.96)	1.35 (1.25-1.45)	1.35 (1.25-1.45)
Retinal detachments and breaks	651 (1.9)	6507 (0.7)	2.68 (2.47-2.90)	1.06 (0.97-1.15)	1.06 (0.98-1.16)
Retinal vascular occlusions	623 (1.8)	3048 (0.3)	5.48 (5.02-5.98)	1.30 (1.19-1.43)	1.30 (1.19-1.43)
Scleritis and episcleritis	256 (0.7)	5080 (0.6)	1.33 (1.17-1.51)	0.83 (0.73-0.95)	0.84 (0.74-0.96)
Dermatological					
Acne	561 (1.6)	84477 (9.3)	0.16 (0.15-0.18)	0.86 (0.79-0.93)	0.87 (0.80-0.95)
Actinic keratosis	3914 (11.4)	25981 (2.9)	4.37 (4.21-4.52)	1.02 (0.98-1.06)	1.03 (0.99-1.07)
Alopecia areata	124 (0.4)	4512 (0.5)	0.73 (0.60-0.86)	0.85 (0.71-1.02)	0.85 (0.71-1.02)
Dermatitis (atopic/ contact/ other unspecified)	10629 (31.0)	235761 (26.0)	1.28 (1.25-1.31)	1.34 (1.31-1.38)	1.35 (1.31-1.38)
Hidradenitis suppurativa	81 (0.2)	3726 (0.4)	0.57 (0.46-0.71)	0.99 (0.78-1.23)	0.97 (0.76-1.20)
Lichen planus	488 (1.4)	6423 (0.7)	2.02 (1.84-2.22)	0.90 (0.81-0.99)	0.90 (0.82-0.99)
Psoriasis	1974 (5.7)	38669 (4.3)	1.37 (1.31-1.44)	1.03 (0.98-1.08)	1.02 (0.98-1.08)
Rosacea	1512 (4.4)	29680 (3.3)	1.36 (1.29-1.44)	0.91 (0.86-0.96)	0.92 (0.87-0.97)
Seborrheic dermatitis	2551 (7.4)	48404 (5.3)	1.42 (1.37-1.48)	1.22 (1.16-1.27)	1.22 (1.17-1.27)
Urticaria	1931 (5.6)	52277 (5.8)	0.98 (0.93-1.02)	1.17 (1.12-1.23)	1.18 (1.12-1.23)
Vitiligo	134 (0.4)	3991 (0.4)	0.89 (0.74-1.05)	0.86 (0.72-1.03)	0.87 (0.72-1.03)
Other					
Down's syndrome	5 (0.0)	604 (0.1)	0.22 (0.08-0.47)	0.52 (0.19-1.15)	0.52 (0.18-1.13)

CI= confidence interval; IMD= Index of Multiple Deprivation; OR= odds ratio.

Supplementary table 12 Prevalence and odds ratio of haematological conditions in adults with atrial fibrillation compared to adults without atrial fibrillation.

Health condition	Adults with AF, n (%) (N=34,338)	Adults without AF, n (%) (N=907,739)	Unadjusted OR (95%CI)	OR adjusted for age and sex (95%CI)	OR adjusted for age, sex and IMD (95%CI)
**Haematological**					
Agranulocytosis	567 (1.7)	6591 (0.7)	2.30 (2.10-2.50)	1.36 (1.24-1.49)	1.36 (1.24-1.49)
Aplastic anaemias	54 (0.2)	342 (0.0)	4.18 (3.10-5.52)	2.07 (1.51-2.81)	2.07 (1.51-2.80)
Folate deficiency anaemia	429 (1.2)	4004 (0.4)	2.86 (2.58-3.15)	1.68 (1.51-1.87)	1.63 (1.46-1.81)
Hyposplenism	186 (0.5)	1513 (0.2)	3.26 (2.79-3.79)	1.83 (1.55-2.15)	1.83 (1.55-2.15)
Immunodeficiencies	34 (0.1)	372 (0.0)	2.42 (1.67-3.38)	2.75 (1.85-3.98)	2.80 (1.88-4.05)
Iron deficiency anaemia	2790 (8.1)	32913 (3.6)	2.35 (2.26-2.45)	1.80 (1.73-1.88)	1.78 (1.71-1.86)
Monoclonal gammopathy of undetermined significance (MGUS)	276 (0.8)	1267 (0.1)	5.80 (5.08-6.59)	1.36 (1.18-1.56)	1.36 (1.19-1.56)
Other anaemias	4236 (12.3)	39490 (4.4)	3.09 (2.99-3.20)	1.91 (1.84-1.98)	1.89 (1.82-1.96)
Other haemolytic anaemias	56 (0.2)	786 (0.1)	1.88 (1.42-2.45)	2.01 (1.49-2.66)	2.01 (1.49-2.66)
Secondary polycythaemia	179 (0.5)	1263 (0.1)	3.76 (3.20-4.39)	1.73 (1.46-2.04)	1.71 (1.44-2.02)
Sickle cell anaemia	11 (0.0)	363 (0.0)	0.80 (0.41-1.39)	1.27 (0.64-2.26)	1.19 (0.60-2.13)
Splenomegaly	210 (0.6)	1909 (0.2)	2.92 (2.52-3.36)	2.15 (1.84-2.51)	2.13 (1.82-2.48)
Thalassaemia	38 (0.1)	1074 (0.1)	0.94 (0.67-1.27)	1.11 (0.78-1.53)	1.10 (0.77-1.52)
Thrombocytopaenia	897 (2.6)	5818 (0.6)	4.16 (3.87-4.46)	2.42 (2.24-2.61)	2.41 (2.23-2.60)
Thrombophilia	90 (0.3)	1785 (0.2)	1.33 (1.07-1.64)	1.77 (1.41-2.20)	1.79 (1.42-2.23)
Venous thromboembolism (excluding pulmonary embolism)	2162 (6.3)	14896 (1.6)	4.03 (3.84-4.22)	1.59 (1.51-1.67)	1.58 (1.50-1.66)
Vitamin B12 deficiency anaemia	1630 (4.7)	15061 (1.7)	2.95 (2.80-3.11)	1.39 (1.31-1.47)	1.37 (1.30-1.45)

CI= confidence interval; IMD= Index of Multiple Deprivation; OR= odds ratio.

Supplementary table 13 Prevalence and odds ratio of benign neoplasms, primary dermatological, haematological and solid organ malignancies, and secondary malignancies, in adults with atrial fibrillation compared to adults without atrial fibrillation.

Health condition	Adults with AF, n (%) (N=34,338)	Adults without AF, n (%) (N=907,739)	Unadjusted OR (95%CI)	OR adjusted for age and sex (95%CI)	OR adjusted for age, sex and IMD (95%CI)
**Benign neoplasm**					
Benign neoplasm and polyp of uterus	569 (1.7)	12062 (1.3)	1.25 (1.15-1.36)	0.77 (0.71-0.84)	0.78 (0.71-0.85)
Benign neoplasm of brain and other parts of central nervous system	198 (0.6)	2356 (0.3)	2.23 (1.92-2.57)	1.11 (0.95-1.29)	1.12 (0.96-1.30)
Benign neoplasm of colon rectum anus and anal canal	3432 (10.0)	32216 (3.5)	3.02 (2.91-3.13)	1.15 (1.11-1.20)	1.15 (1.11-1.20)
Benign neoplasm of ovary	691 (2.0)	25460 (2.8)	0.71 (0.66-0.77)	0.83 (0.77-0.90)	0.83 (0.77-0.90)
Benign neoplasm of stomach and duodenum	1050 (3.1)	8562 (0.9)	3.31 (3.10-3.53)	1.16 (1.08-1.24)	1.16 (1.08-1.24)
Fibromatoses	1334 (3.9)	11283 (1.2)	3.21 (3.03-3.40)	0.91 (0.86-0.97)	0.91 (0.86-0.97)
Haemangioma any site	686 (2.0)	13684 (1.5)	1.33 (1.23-1.44)	1.07 (0.99-1.16)	1.09 (1.00-1.18)
Leiomyoma of uterus	469 (1.4)	15984 (1.8)	0.77 (0.70-0.85)	0.54 (0.49-0.59)	0.54 (0.49-0.59)
Nasal polyp	815 (2.4)	10102 (1.1)	2.16 (2.01-2.32)	1.05 (0.98-1.14)	1.06 (0.98-1.14)
Primary dermatological malignancy					
Primary malignancy malignant melanoma	456 (1.3)	4951 (0.5)	2.45 (2.23-2.70)	0.96 (0.87-1.06)	0.97 (0.88-1.08)
Primary malignancy other skin and subcutaneous tissue	3877 (11.3)	24299 (2.7)	4.63 (4.46-4.80)	1.10 (1.06-1.15)	1.11 (1.07-1.15)
Primary haematological malignancy					
Hodgkin lymphoma	47 (0.1)	628 (0.1)	1.98 (1.45-2.63)	1.65 (1.19-2.24)	1.67 (1.20-2.26)
Leukaemia	193 (0.6)	1442 (0.2)	3.55 (3.05-4.12)	1.37 (1.16-1.60)	1.37 (1.16-1.60)
Multiple myeloma and malignant plasma cell neoplasms	98 (0.3)	509 (0.1)	5.10 (4.09-6.30)	1.42 (1.12-1.77)	1.42 (1.12-1.77)
Myelodysplastic syndromes	118 (0.3)	372 (0.0)	8.41 (6.81-10.31)	1.97 (1.58-2.45)	1.97 (1.58-2.45)
Non-Hodgkin lymphoma	254 (0.7)	1980 (0.2)	3.41 (2.98-3.88)	1.23 (1.07-1.41)	1.23 (1.07-1.41)
Polycythaemia vera	160 (0.5)	1029 (0.1)	4.13 (3.48-4.86)	1.67 (1.39-1.99)	1.66 (1.38-1.98)
Primary solid organ malignancy					
Primary malignancy biliary tract	36 (0.1)	274 (0.0)	3.48 (2.41-4.85)	1.04 (0.71-1.47)	1.04 (0.71-1.47)
Primary malignancy bladder	386 (1.1)	1878 (0.2)	5.48 (4.91-6.11)	1.16 (1.03-1.30)	1.16 (1.03-1.30)
Primary malignancy bone and articular cartilage	18 (0.1)	195 (0.0)	2.44 (1.45-3.84)	2.04 (1.18-3.34)	2.04 (1.18-3.35)
Primary malignancy brain, other central nervous system and intracranial	22 (0.1)	418 (0.0)	1.39 (0.88-2.08)	1.39 (0.86-2.13)	1.39 (0.86-2.14)
Primary malignancy breast	1045 (3.0)	12959 (1.4)	2.17 (2.03-2.31)	0.84 (0.78-0.90)	0.84 (0.79-0.90)
Primary malignancy cervical	34 (0.1)	791 (0.1)	1.14 (0.79-1.58)	0.73 (0.51-1.03)	0.72 (0.49-1.01)
Primary malignancy colorectal and anus	962 (2.8)	5598 (0.6)	4.64 (4.33-4.98)	1.15 (1.07-1.24)	1.15 (1.07-1.24)
Primary malignancy kidney and ureter	280 (0.8)	1861 (0.2)	4.00 (3.52-4.53)	1.23 (1.08-1.41)	1.23 (1.08-1.40)
Primary malignancy liver	51 (0.1)	388 (0.0)	3.48 (2.57-4.61)	1.07 (0.78-1.45)	1.07 (0.78-1.44)
Primary malignancy lung and trachea	198 (0.6)	993 (0.1)	5.30 (4.53-6.16)	1.47 (1.25-1.73)	1.46 (1.24-1.71)
Primary malignancy mesothelioma	<5	<5	NA (NA-NA)	NA (NA-NA)	NA (NA-NA)
Primary malignancy oesophageal	106 (0.3)	355 (0.0)	7.91 (6.34-9.80)	2.14 (1.69-2.70)	2.14 (1.69-2.69)
Primary malignancy oropharyngeal	137 (0.4)	1211 (0.1)	3.00 (2.50-3.56)	1.07 (0.88-1.28)	1.06 (0.88-1.27)
Primary malignancy other	535 (1.6)	3589 (0.4)	3.99 (3.63-4.36)	1.29 (1.17-1.42)	1.29 (1.17-1.42)
Primary malignancy ovarian	120 (0.3)	1575 (0.2)	2.02 (1.67-2.42)	0.96 (0.79-1.16)	0.96 (0.79-1.16)
Primary malignancy pancreatic	29 (0.1)	129 (0.0)	5.95 (3.90-8.76)	1.73 (1.11-2.62)	1.73 (1.11-2.61)
Primary malignancy prostate	1665 (4.8)	9881 (1.1)	4.63 (4.39-4.88)	0.89 (0.84-0.94)	0.89 (0.84-0.95)
Primary malignancy stomach	49 (0.1)	200 (0.0)	6.48 (4.69-8.78)	1.63 (1.16-2.25)	1.63 (1.16-2.25)
Primary malignancy testicular	35 (0.1)	785 (0.1)	1.18 (0.82-1.63)	0.87 (0.60-1.22)	0.88 (0.61-1.23)
Primary malignancy thyroid	67 (0.2)	922 (0.1)	1.92 (1.49-2.44)	1.35 (1.03-1.74)	1.35 (1.03-1.74)
Primary malignancy uterine	145 (0.4)	1237 (0.1)	3.11 (2.61-3.68)	1.04 (0.87-1.25)	1.04 (0.87-1.24)

Supplementary table 13 (continued).

Health condition	Adults with AF, n (%) (N=34,338)	Adults without AF, n (%) (N=907,739)	Unadjusted OR (95%CI)	OR adjusted for age and sex (95%CI)	OR adjusted for age, sex and IMD (95%CI)
**Secondary malignancy**					
Secondary malignancy adrenal gland	41 (0.1)	492 (0.1)	2.20 (1.58-2.99)	0.68 (0.48-0.93)	0.67 (0.48-0.92)
Secondary malignancy bone	468 (1.4)	3375 (0.4)	3.70 (3.36-4.08)	1.07 (0.97-1.19)	1.07 (0.97-1.19)
Secondary malignancy bowel	37 (0.1)	293 (0.0)	3.34 (2.33-4.64)	1.24 (0.85-1.76)	1.24 (0.85-1.75)
Secondary malignancy brain, central nervous system	84 (0.2)	969 (0.1)	2.29 (1.82-2.85)	0.88 (0.69-1.10)	0.87 (0.69-1.09)
Secondary malignancy liver bile duct	391 (1.1)	3576 (0.4)	2.91 (2.62-3.23)	0.94 (0.84-1.04)	0.93 (0.84-1.04)
Secondary malignancy lung	310 (0.9)	2664 (0.3)	3.10 (2.75-3.48)	1.04 (0.92-1.17)	1.04 (0.91-1.17)
Secondary malignancy lymph nodes	18 (0.1)	228 (0.0)	2.09 (1.25-3.27)	0.82 (0.49-1.32)	0.83 (0.49-1.33)
Secondary malignancy other organs	395 (1.2)	3147 (0.3)	3.35 (3.01-3.71)	1.14 (1.02-1.27)	1.14 (1.02-1.27)
Secondary malignancy pleura	86 (0.3)	696 (0.1)	3.27 (2.60-4.07)	1.11 (0.87-1.39)	1.11 (0.87-1.39)
Secondary malignancy retroperitoneum and peritoneum	148 (0.4)	1592 (0.2)	2.46 (2.07-2.91)	0.94 (0.78-1.12)	0.94 (0.78-1.11)

CI= confidence interval; IMD= Index of Multiple Deprivation; OR= odds ratio.

Supplementary table 14 Prevalence and odds ratio of infectious diseases in adults with atrial fibrillation compared to adults without atrial fibrillation.

Health condition	Adults with AF, n (%) (N=34,338)	Adults without AF, n (%) (N=907,739)	Unadjusted OR (95%CI)	OR adjusted for age and sex (95%CI)	OR adjusted for age, sex and IMD (95%CI)
Aspiration pneumonitis	1285 (3.7)	4015 (0.4)	8.75 (8.21-9.32)	2.26 (2.11-2.42)	2.25 (2.10-2.41)
Bacterial diseases (excluding tuberculosis)	9139 (26.6)	53800 (5.9)	5.76 (5.61-5.90)	2.64 (2.57-2.71)	2.62 (2.55-2.70)
Ear and upper respiratory tract infections	125 (0.4)	3863 (0.4)	0.85 (0.71-1.02)	1.63 (1.34-1.95)	1.61 (1.33-1.93)
Human immunodeficiency virus (HIV)	12 (0.0)	952 (0.1)	0.33 (0.18-0.56)	0.39 (0.21-0.66)	0.37 (0.20-0.64)
Infection of anal and rectal regions	15 (0.0)	559 (0.1)	0.71 (0.41-1.14)	1.09 (0.62-1.80)	1.06 (0.60-1.75)
Infection of bones and joints	94 (0.3)	530 (0.1)	4.70 (3.75-5.82)	2.18 (1.71-2.75)	2.15 (1.69-2.72)
Infection of liver	61 (0.2)	413 (0.0)	3.91 (2.96-5.07)	1.50 (1.12-1.98)	1.48 (1.10-1.95)
Infection of male genital system	126 (0.4)	1157 (0.1)	2.89 (2.39-3.45)	1.79 (1.46-2.17)	1.77 (1.45-2.15)
Infection of other or unspecified genitourinary system	137 (0.4)	3062 (0.3)	1.18 (0.99-1.40)	2.68 (2.22-3.20)	2.58 (2.14-3.08)
Infection of skin and subcutaneous tissues	230 (0.7)	4406 (0.5)	1.38 (1.21-1.58)	2.40 (2.07-2.76)	2.36 (2.04-2.71)
Infections of the digestive system	302 (0.9)	3780 (0.4)	2.12 (1.88-2.38)	1.30 (1.15-1.47)	1.28 (1.12-1.44)
Infections of the heart	198 (0.6)	543 (0.1)	9.69 (8.21-11.38)	7.40 (6.09-8.98)	7.37 (6.06-8.94)
Lower respiratory tract infections	7559 (22.0)	32827 (3.6)	7.52 (7.32-7.73)	2.84 (2.76-2.93)	2.84 (2.75-2.93)
Mycoses	59 (0.2)	420 (0.0)	3.72 (2.80-4.84)	1.80 (1.33-2.40)	1.78 (1.32-2.37)
Nervous system infection	174 (0.5)	1541 (0.2)	3.00 (2.55-3.49)	2.04 (1.72-2.41)	2.02 (1.70-2.39)
Other infection	10945 (31.9)	69300 (7.6)	5.66 (5.53-5.80)	3.18 (3.10-3.26)	3.16 (3.08-3.24)
Parasitic infections	34 (0.1)	628 (0.1)	1.43 (0.99-1.99)	1.75 (1.20-2.49)	1.71 (1.16-2.42)
Peritonitis	781 (2.3)	8690 (1.0)	2.41 (2.23-2.59)	1.41 (1.30-1.53)	1.40 (1.30-1.51)
Rheumatic fever	441 (1.3)	1731 (0.2)	6.81 (6.12-7.56)	2.21 (1.97-2.47)	2.21 (1.97-2.47)
Septicaemia	27 (0.1)	243 (0.0)	2.94 (1.93-4.29)	4.36 (2.75-6.71)	4.32 (2.72-6.65)
Tuberculosis	586 (1.7)	5628 (0.6)	2.78 (2.55-3.03)	1.18 (1.07-1.29)	1.17 (1.06-1.27)
Urinary tract infections	8453 (24.6)	40778 (4.5)	6.94 (6.76-7.13)	2.57 (2.50-2.65)	2.56 (2.49-2.63)
Viral diseases (excluding chronic hepatitis and HIV)	636 (1.9)	6312 (0.7)	2.70 (2.48-2.92)	2.70 (2.47-2.95)	2.66 (2.43-2.91)

CI= confidence interval; IMD= Index of Multiple Deprivation; OR= odds ratio.
